# A Guide to the Practical Examination of Urine

**Published:** 1886-02

**Authors:** 


**A Guide to the Practical Examination of Urine.** For the use of Physicians and Students. By JAMES TYSON, M. D., Prof. of General Pathology and Morbid Anatomy in the University of Pennsylvania, Physician to the Philadelphia Hospital, etc. Fifth edition; revised and corrected, with colored plates and wood engravings. Philadelphia: P. Blakiston, Son & Co., 1012 Walnut street. 1886.

We have always thought well of Tyson's work on this subject, and this fifth edition increases our good opinion of it. Among the multitude of manuals upon urinalysis, we have often hesitated which to commend to inquiring physicians. "The new edition of Tyson," will be our reply now, at least, till something better comes out. The only fault we would find with it is, that it is growing in size. In a manual which aims to give clear and readily comprehensible directions for ordinary examinations of urine, conciseness and brevity are essential features. However, we find little in this edition we would wish eliminated.

